# Lipectomy associated to obesity produces greater fat accumulation in the visceral white adipose tissue of female compared to male rats

**DOI:** 10.1186/s12944-019-0988-5

**Published:** 2019-02-09

**Authors:** Fábio da Silva Pimenta, Hadnan Tose, Élio Waichert Jr, Márcia Regina Holanda da Cunha, Fabiana Vasconcelos Campos, Elisardo Corral Vasquez, Hélder Mauad

**Affiliations:** 1 Department of Physiological Sciences, Health Sciences Center, Federal University of Espírito Santo, Av. Marechal Campos 1468, Vitória, ES CEP 29042-751 Brazil; 2 Laboratory of Translational Physiology and Pharmacology, Pharmaceutical Sciences Graduate Program, Vila Velha University (UVV), Avenida Comissário José Dantas de Melo, 21 Bairro Boa Vista II, Vila Velha, ES CEP 29102-920 Brazil; 3 Departament of Medical Clinic, Escola Superior de Ciências da Santa Casa de Misericórdia de Vitória (EMESCAM), Av. Nossa Sra. da Penha, 2190 - Bela Vista, Vitória, ES CEP 29027-502 Brazil; 4 Faculdade Estácio, Av. Dr. Herwan Modenese Wanderley, 1001. Bairro Jardim Camburi, Vitória, ES CEP 29092-095 Brazil; 5 Sports Department, Center for Physical Education and Sports, Federal University of Espírito Santo, Avenida Fernando Ferrari, 514. Bairro Goiabeiras, Vitória, ES CEP 29075-910 Brazil

**Keywords:** Obesity, Lipectomy, White adipose tissue, Monosodium glutamate

## Abstract

**Background:**

Mobility of fat deposited in adipocytes among different fatty territories can play a crucial role in the pathogenesis of obesity-related diseases. Our goal was to investigate which of the remaining fat pads assume the role of accumulating lipids after surgical removal of parietal WAT (lipectomy; LIPEC) in rats of both sexes displaying MSG-induced obesity.

**Methods:**

The animals entered the study straight after birth, being separated according to gender and randomly divided into CON (control, saline-treated) and MSG (monosodium glutamate-treated) groups. Next, the animals underwent LIPEC or sham-operated surgery (SHAM). Obesity was induced by the injection of MSG (4 mg/g/day) during neonatal stage (2nd to 11th day from birth). LIPEC was performed on the 12th week, consisting in the withdrawal of parietal WAT. On the 16th week, the following WATs were isolated and collected: peri-epididymal-WAT (EP-WAT); parametrial-WAT (PM-WAT); omental-WAT (OM-WAT); perirenal-WAT (PR-WAT) and retroperitoneal-WAT (RP-WAT).

**Results:**

The adiposity index was significantly increased in both male (3.2 ± 0.2** vs 1.8 ± 0.1) and female (4.9 ± 0.7* vs 2.6 ± 0.3) obese rats compared to their respective control groups. LIPEC in obese animals produced fat accumulation in visceral fat sites in a more accentuated manner in female (3.6 ± 0.3** vs 2.8 ± 0.3 g/100 g) rather than in male (1.8 ± 0.2* vs 1.5 ± 0.1 g/100 g) rats compared to obese non-lipectomized animals. Among the visceral WATs, the greater differences were observed between gonadal WATs of obese lipectomized rats, with higher accumulation having been observed in PM-WAT (2.8 ± 0.3* vs 2.1 ± 0.2 g/100 g) rather than in EP-WAT (1.0 ± 0.1 ± 0.9 ± 0.1 g/100 g) when compared to obese non-lipectomized animals.

**Conclusions:**

The results of the present study led us to conclude that obesity induced by MSG treatment occurs differently in male and female rats. When associated with parietal LIPEC, there was a significant increase in the deposition of visceral fat, which was significantly higher in obese female rats than in males, indicating that fat mobility among WATs in lipectomized-obese rats can occur more expressively in particular sites of remaining WATs.

## Background

Obesity has become a major public health challenge [1], for the prevalence of overweight and obese people has been increasing worldwide, being related to alarming epidemiological risk factors for diabetes, cardiovascular disease, cancer and premature death [2]. The genesis of obesity and its physiological consequences have been extensively studied. Our particular interest lies in understanding the mobility of fat deposited in adipocytes among different fatty territories, which could play a crucial role in the pathogenesis of obesity-related diseases.

Specific conditions such as gender, blood circulation, neurological factors and obesity itself could account for the local differences observed among different fat depots regarding cellular composition, size and function, making them act as “separate miniorgans” [3].

It is well known that the fat stored in adipocytes is not homogeneous. Its distribution among the different white adipose tissue (WAT) depots affects the production of inflammatory cytokines and chemokines by pre-adipocytes, which is also influenced by age and body weight [3–5]. The bulk of the body’s fat resides in subcutaneous WAT depots, which can be distinctive due to unique metabolic and physiological characteristics. Also known as parietal WAT, these subcutaneous fat depots are located mainly in the inferior portion of the body, where they protect the muscles from fat infiltration by capturing the excess of lipids, contributing also to glucose homeostasis as metabolic dissipators [6]. On the other hand, it is widely known that upper-body adiposity constitutes an important risk factor for many diseases, with visceral adiposity playing a major role in metabolic disorders, including insulin resistance, hyperinsulinemia, type-2 diabetes mellitus, among others [7–10]. Therefore, knowing the precise location and amount of the different fat depots in one’s body is critical for the comprehension of the actual role of regional adiposity as a risk factor in epidemiological studies [11].

Due to culturally-defined beauty standards, surgical removal of WAT through liposuction has been widely employed. It consists in the withdrawal of accumulation and/or bad distribution areas of parietal WAT, mainly from the deep layer (lamellar). This procedure has also been employed experimentally through parietal LIPEC, presenting controversial results concerning compensatory increases of WAT and the mobility of lipids among fat territories. This controversy is observed in both experimental [12, 13] and clinical studies [14–17]. Strong evidence points to changes taking place in the parietal WAT after its resection, including fat deposition in non-lipectomized areas. However, which are these areas, how do they behave after parietal LIPEC and are these issues influenced by gender are questions that remain to be fully answered.

In order to assess these issues, we employed an experimental model in which obesity was induced by treating neonatal rats with monosodium glutamate (MSG). MSG provokes damage in the central nervous system (CNS), predominantly in the hypothalamus arcuate nucleus (HAN), which is highly involved in the release of neurotransmitters, peptides and hormones [18–21]. HAN lesions occurring during the neonatal period lead to several neuro-endocrine changes in the regulation of food intake in rats, resulting in decreased naso-anal length, hyperlipidemia and, above all, obesity [22, 23]. In addition, it has been reported that adult rats treated with MSG displayed hyperinsulinemia, pointing to an impairment of insulin sensitivity [24].

Despite the many results achieved so far on the connection between MSG treatment and obesity, the occurrence of fat mobility among WATs in this experimental model remains to be addressed, particularly following LIPEC in obese rats. Thus, the aim of this study was to investigate which of the remaining fat pads assume the key role of accumulating lipids after surgical removal of parietal WAT in rats of both sexes displaying MSG-induced obesity.

## Methods

### Animal models and ethical issues

Male and female Wistar rats – included in the study straight after birth – were maintained on a standard diet and tap water, being exposed to a 12/12 h light-dark cycle at 23^o^ C. First, the animals were separated according to gender and randomly divided into the following groups: CON (control, saline-treated, male/female *n* = 11/10, respectively) and MSG (monosodium glutamate-treated male/female *n* = 15/16, respectively). Next, the animals underwent (LIPEC) or sham-operated surgery (SHAM), comprising the following groups: males CON-LIPEC (*n* = 4), CON-SHAM (*n* = 7), MSG-LIPEC (*n* = 10) and MSG-SHAM (*n* = 5); females CON-LIPEC (*n* = 5), CON-SHAM (*n* = 5), MSG-LIPEC (*n* = 11) and MSG-SHAM (*n* = 5).

All experiments were conducted in accordance with the U.S. National Institute of Health Guidelines for the Care and Use of Laboratory Animals, and study protocols were previously approved by our Institutional Ethics Committee for the Use of Animals (Protocol # 034/2011).

### Obesity-inducing treatment

Obesity was induced by subcutaneous injection of MSG (4 mg/g/day) during neonatal stage (2nd to 11th days from birth). Control animals were submitted to the same schedule of injections, receiving NaCl hypertonic solution (1.45%) instead. At the age of 21 days, animals were weaned and placed in polypropylene boxes. All animals received the same standard diet ad libitum throughout the study.

### Surgical procedures

LIPEC was performed on the 12th week under anesthesia with chloral hydrate 10%, intraperitoneally. With the animals positioned in dorsal decubitus, the skin of the inguinal region and a small notch from the midpoint of the thigh to the knee joint of the animals were pinched. Then, a divulsion was performed for the detachment of WAT from the aponeurotic structures and the skin, and also for the detachment of the adipose projection on the flanks until the reflection between the ventral abdomen and the back-lobe and posterior withdrawal of parietal WAT. The skin was then sutured with 4–0 cotton yarn, by continuous scrubbing. Removal of the parietal WAT was done bilaterally and weighed on a precision scale. Sham surgery consisted in surgical incision and cotton suture only.

On the 16th week, the WATs were isolated and collected from the following sites: parietal WAT (PT-WAT), only in animals non-lipectomized at the 12th week; perigonadal-WATs: peri-epididymal-WAT (EP-WAT) and parametrial-WAT (PM-WAT); omental-WAT (OM-WAT), which corresponds to the greater omentum; perirenal-WAT (PR-WAT) and retroperitoneal-WAT (RP-WAT).

Firstly, animals were decapitated and submitted to tricotomy in the abdominal anterior region and antero-medial rear paws. Next, animals were fixed in a surgical board in supine position. The individual procedures for the removal of each particular WAT are briefly described below:

#### Parietal-WAT

This procedure – performed only in sham groups (CON-SHAM and MSG-SHAM) – was conducted as described above for the animals lipectomized at the 12th week.

#### Perigonadal-WATs

Initially, for male rats, a medium pubic xiphoid opening and a perigonadal adipose cushion were clamped. This tissue was then pulled for testicular exposure and individualization of peri-epididimal fat planes. In female rats, the uterus was pulled and sections of parametrial-WAT projections were performed.

#### Omental-WATs

This tissue was collected after traction of the spleen and the stomach, exposing the greater curvature of the latter for resection of the greater omentum.

#### Perirenal-WAT

It was collected by bouncing the intestines laterally for visualization of the kidney and its perirenal fat. By tractioning the kidney, the limits of this fat pad are exposed and excised. This procedure was performed bilaterally.

#### Retroperitoneal-WAT

After renal resection and subsequent removal of the perirenal-WAT and the adrenal gland, retroperitoneal fat is easily visualized. It was resected from the pelvic region up to below the diaphragm insertion, bilaterally.

### Data analysis

Considering the variation in the total body weight of the animals, all the WATs obtained (g) were normalized by 100 g of body weight. The sum of all WATs – but the parietal-WAT that was removed in the 12th week – is referred to as “total fats”. The Adiposity Index was calculated by the sum of total fats divided by corporal weight and multiplied by 100, representing the amount of fat from all WATs aforementioned by grams of animal. The term “Visceral WAT” refers to the quantity of fat accumulated in the viscera. It comprises the sum of omental-, perirenal and epididymal−/parametrial-WATs. Extra-Visceral WAT represents the fat accumulated in the abdomen, but not in the viscera. It was obtained by the sum of retroperitoneal- and parietal-WATs, except in those cases where parietal fat was removed.

### Statistical analysis

The results are expressed as the mean ± SEM. For the evaluation of the normality of the data distribution we used the D’Agostino and Pearson test. In addition, all data were submitted to two-way analysis of variance (ANOVA) followed by Tukey test, and, for some of the data, to the Student’s t-test. Statistical significance was achieved when *p* < 0.05.

## Results

Anthropometric parameters of the different groups studied are shown in Table 1. After 16 weeks, both MSG-treated groups presented body weight values smaller than those of CON groups, which was true for both sexes (although for sham females the change was not statistically significant). On the other hand, we observed that the LIPEC procedure did not induce changes in body weight in either CON or MSG-treated animals, which, again, was true for males and females. It is worthy of note that female animals weighted less than males to begin with. These results clearly show that MSG treatment induced less body weight gain in both male and female animals, with LIPEC having no effects on this parameter.

**Table 1:** Anthropometrics parameters of animals

	CON-SHAM	CON-LIPEC	MSG-SHAM	MSG-LIPEC
	*Male*			
Body Weight (g)	380 ± 16	412 ± 4	275 ± 6**	294 ± 12^##^
Total Fat (g)	6.8 ± 0.7	7.3 ± 0.7	8.9 ± 0.8	9.9 ± 0.5^#^
Adiposity Index	1.8 ± 0.1	1.8 ± 0.2	3.2 ± 0.2**	3.2 ± 0.2^#^
	*Female*			
Body Weight (g)	244 ± 6	244 ± 4	203 ± 13	185 ± 4^##^
Total Fat (g)	6.3 ± 0.7	5.2 ± 0.3**	9.3 ± 1.2*	10.2 ± 1.1^##††^
Adiposity Index	2.6 ± 0.3	2.1 ± 0.2	4.9 ± 0.7*	5.5 ± 0.5^##^

*p < 0.05 e **p < 0.01, different to CON-SHAM group. ^#^*p* < 0.05 e ^##^*p* < 0.01, different to CON-LIPEC group. ^†^*p* < 0.05 e ^††^*p* < 0.01, different to MSG-SHAM group. ANOVA 2 way, post hoc: Tukey

In order to evaluate actual fat accumulation in the animals from all groups, we determined the parameter total fat (Table 1). We observed that, while LIPEC significantly increased the total fat in MSG-LIPEC male rats when compared to CON-LIPEC animals, no significant differences were observed regarding this parameter when animals belonging to MSG-LIPEC and MSG-SHAM groups were compared between themselves, as well as between CON-LIPEC and CON-SHAM groups. On the other hand, LIPEC induced opposite effects regarding total fat accumulation in CON and MSG-treated female rats: while the procedure led to smaller total fat values in CON animals, significantly higher values were found in MSG-treated rats. In fact, total fat values in MSG- LIPEC female rats were significantly higher than those of either MSG-SHAM and CON- LIPEC animals. These results clearly show that there is greater accumulation of total fat in obese female rats when compared to males, and that LIPEC potentiated this accumulation.

Considering that the procedures employed, i.e., LIPEC and MSG treatment, produced different changes in the body weight parameter, we have also analyzed their effects on the adiposity index of the animals (Table 1), which permitted us to compare values of fat per gram of corporal weight among the groups. We observed that MSG-treated male rats – LIPEC or not – presented significantly higher adiposity indexes when compared to their respective controls, which was also true for female rats. Taken together, these results indicate that MSG-induced obesity led to a more pronounced fat accumulation in females, with this accumulation being even higher when obesity was associated with LIPEC.

**Fig. 1:**
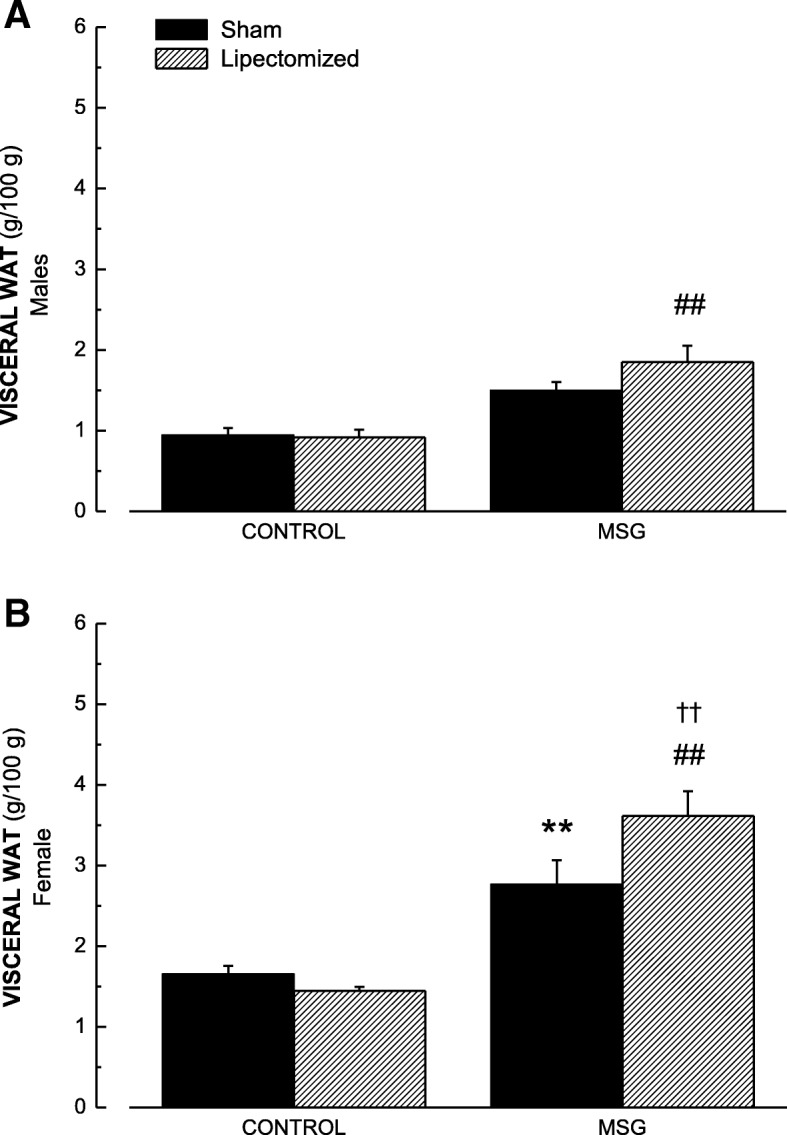
Values of Visceral Fat in CON-LIPEC, CON-SHAM, MSG-LIPEC and MSG-SHAM) groups in male (**a**) and female (**b**) rats. **p < 0.01 compared to respective CON-SHAM. ^##^*p* < 0.01 compared to CON-LIPEC. ^††^*p* < 0.01 compared to MSG-SHAM

We then asked ourselves how the distribution of fat in different corporal regions occurs after LIPEC, in both control and obese animals. To answer that, we separated groups of WATs based on their anatomical localization and functional aspects. To begin with, we considered the group deemed visceral WAT (Fig. 1). We observed that, in males, while there was a significant increase in visceral WAT in obese animals submitted to LIPEC when compared to lipectomized control animals, obesity alone did not have a significant effect (Fig. 1a). On the other hand, in female animals, we observed that obesity alone produced a significant increase in visceral WAT when compared to the sham control group. Moreover, obesity associated to LIPEC induced significantly higher increases in visceral WAT not only compared to lipectomized control animals but also to no LIPEC obese ones (Fig. 1b). These data strongly indicate that the effects of LIPEC on visceral WAT accumulation are much more severe in obese female rats than in obese males.

**Fig. 2:**
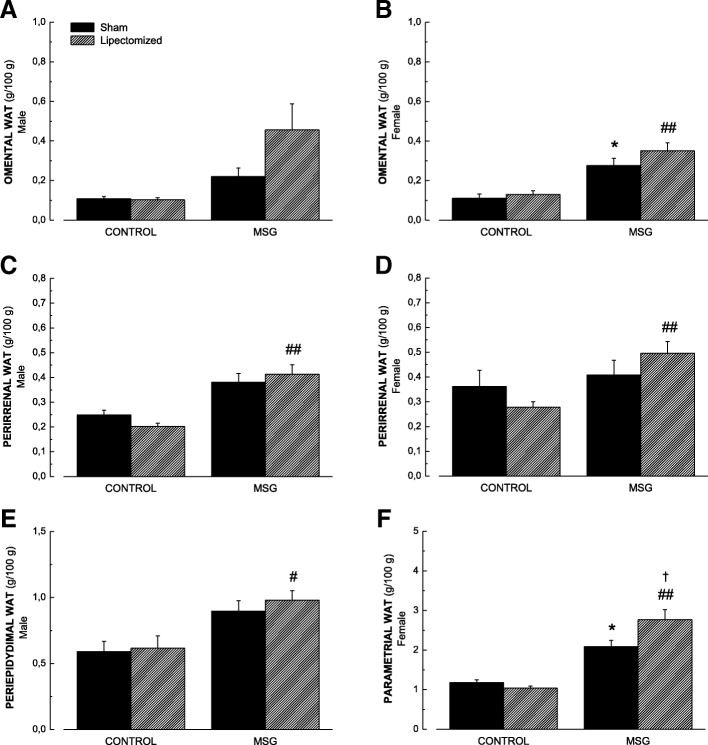
Values of Omental WAT (**a** and **b**), Perirenal WAT (**c** and **d**) and Gonadal (Periepidydimal and Parametrial) WATs (**e** and **f**) in CON-LIPEC, CON-SHAM, MSG-LIPEC and MSG-SHAM) groups in male and female rats. *p < 0.05 and **p < 0.01 compared to respective CON-SHAM. ^#^*p* < 0.05 and ^##^*p* < 0.01 compared to CON-LIPEC. ^†^*P* < 0.05 compared to MSG-SHAM

Next, we assessed the individual contribution of each one of the WATs that compose the visceral fat (Fig. 2). We did not observe significant differences regarding the Omental-WAT in male rats (Fig. 2a). In females, we observed increased fat accumulation in both obesity-induced groups when compared to their respective controls (Fig. 2b).

As to Perirenal-WAT, we observed, for both sexes, that lipectomized obese animals presented higher fat accumulation values when compared to lipectomized control animals, with obesity by itself showing no significant effects (Fig. 2).

Finally, regarding Gonadal-WATs, we observed increased values of Periepididymal WAT in lipectomized obese animals when compared to their lipectomized controls (Fig. 2e). Parametrial WAT values, on the other hand, were found to be significantly higher in both MSG groups when compared to their respective controls, as well as when obese lipectomized animals were compared to obese non-lipectomized animals (Fig. 2f). Taken together, these results suggest that the higher accumulation of visceral WATs observed in female rats is mainly due to an increase in Parametrial WAT in obese animals, particularly in those submitted to LIPEC.

We have also dwelt upon the differences between extravisceral and extraabdominal fat accumulation, through the analysis of retroperitoneal- and parietal-WATs (Table 2). We observed that, for both sexes, obesity itself induced higher retroperitoneal and parietal fat accumulation when compared to control animals. In obese female rats, LIPEC induced a significantly higher accumulation of retroperitoneal fat, clearly indicating that post-LIPEC fat mobility to this pad is gender-dependent. It is important to point out that, as in both lipectomized groups the parietal values are relative to the 12th week, while in sham-operated groups these values are relative to the 16th week, one should indeed expect no significant differences among animals undergoing the procedure with regards to their respective controls.

**Table 2:** Retroperitoneal (Extravisceral) and Parietal (Extraabdominal) WATs of animals

	CON-SHAM	CON-LIPEC	MSG-SHAM	MSG-LIPEC
WAT (g/100 g)	*Male rats*			
Retroperitoneal	0.84 ± 0.06	0.84 ± 0.07	1.72 ± 0.17**	1.34 ± 0.14
Parietal	1.17 ± 0.06	1.14 ± 0.10	2.48 ± 0.28**	3.27 ± 0.19
	*Female rats*			
Retroperitoneal	0.91 ± 0.15	0.69 ± 0.05	1.66 ± 0.16*	1.84 ± 0.23^##^
Parietal	0.97 ± 0.07	0.95 ± 0.06	3.11 ± 0.28*	3.57 ± 0.68

*p < 0.05 e **p < 0.01, different to CON-SHAM group. ^##^*p* < 0.01, different to CON-LIPEC group. ANOVA 2 way, post hoc: Tukey

**Fig. 3:**
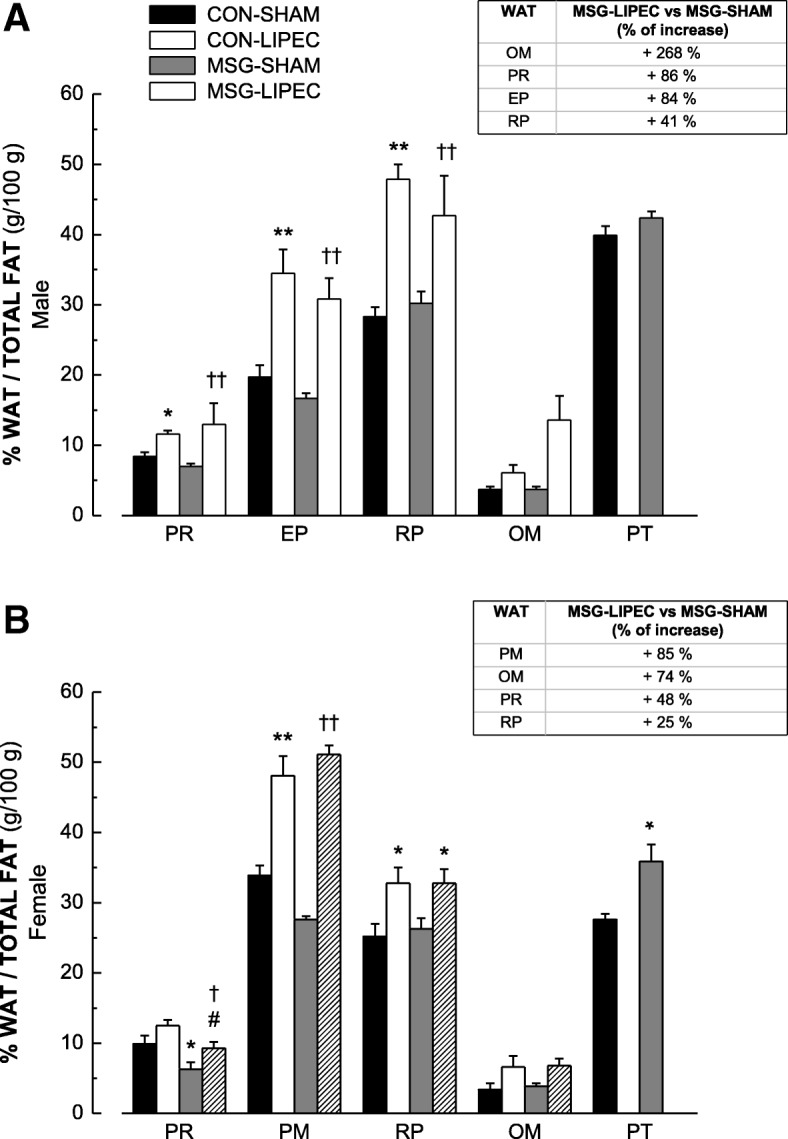
Percentage of WAT in relation to total fat in CON-LIPEC, CON-SHAM, MSG-LIPEC and MSG-SHAM) groups in male (**a**) and female (**b**) rats. *p < 0.05 and **p < 0.01 compared to respective CON-SHAM. ^#^*p* < 0.05 and ^##^*p* < 0.01 compared to CON-LIPEC. ^†^*P* < 0.05 and ^††^*p* < 0.01 compared to MSG-SHAM. WATs: PT, parietal; RP, retroperitoneal; EP, periepidydimal; PM, parametrial; PR, perirenal; OM, omental. Tables indicate percentage of increasing of WATs between MSG-LIPEC and MSG-SHAM groups

The mobility of fat among WATs after MSG-induced obesity alone or associated to the removal of parietal WAT through LIPEC was then evaluated. In order to assess this parameter, we first calculated the relative percentage of each WAT collected at the 16th week in relation to normalized values of total fat from the same animal, except for parietal-WAT, which was considered only in non-lipectomized animals. First of all, we observed that in male rats (Fig. 3a) obesity alone (MSG-SHAM) did not yield significant changes in fat accumulation among the different territories. On the other hand, LIPEC alone (CON-LIPEC) was able to drive major accumulation of fat in perirenal, periepidydimal and retroperitoneal WATs compared to non-lipectomized control animals (CON-SHAM). When associated to obesity (MSG-LIPEC vs MSG-SHAM), we observed that LIPEC promoted crescent accumulation of fat among the following WATs: omental (268%) > > perirenal (86%) > periepidydimal (84%) > retroperitoneal (41%). These results indicate that while LIPEC, alone or associated to obesity, promotes proportionally distinct levels of fat accumulation in different WATs, obesity alone – in spite of inducing higher adiposity – led to fat distribution in a more uniform manner.

In female rats (Fig. 3b), we found that obesity alone led to proportionally higher levels of fat being distributed to perirenal and parietal WATs. LIPEC alone promoted a major percentage of fat accumulation in parametrial and retroperitoneal WATs when compared to non-lipectomized control animals. And, unlike in male rats, the effect of LIPEC associated to obesity yielded the following crescent proportional accumulation: parametrial (85%) > omental (74%) > perirenal (48%) > retoperitoneal (25%), corroborating the results in which we observed major increased accumulation in visceral fat.

**Fig. 4:**
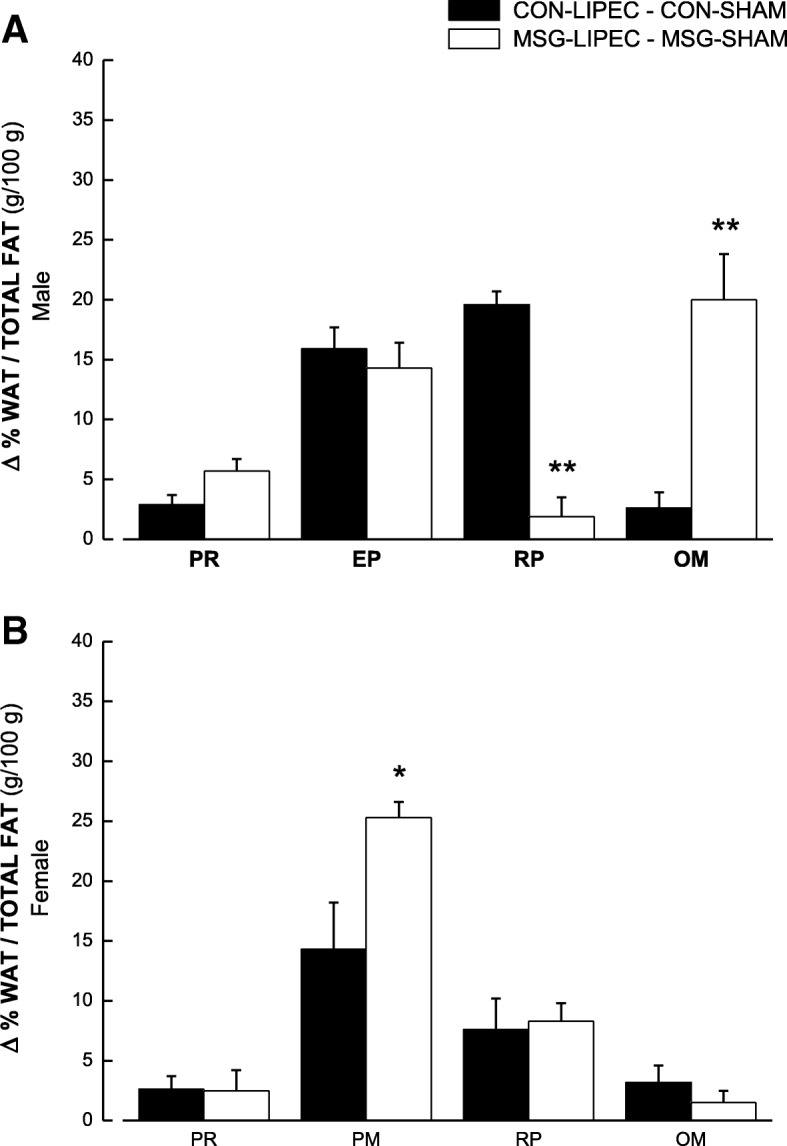
Percentage changes of WAT in relation to total fat between control (CON-LIPEC *minus* CON-SHAM) and MSG (MSG-LIPEC *minus* MSG-SHAM) groups in male (**a**) and female (**b**) rats. *p < 0.05 and **p < 0.01 in relation to respective control group. WATs: RP, retroperitoneal; EP, periepidydimal; PM, parametrial; PR, perirenal; OM, omental

Finally, we analyzed the magnitude of the effects induced by LIPEC in both obese (MSG) and non-obese (control) groups (Fig. 4). We have done that by computing the difference in percentage of WATs in relation total fat between lipectomized and non-lipectomized animals. In male rats (Fig. 4a), we observed that LIPEC induced similar accumulation of fat in perirenal and periepidydimal WATs in both obese and non-obese animals, with the retroperitoneal WAT in obese rats being significantly decreased by the procedure. The exact opposite was observed with regards to omental WAT, which was significantly increased by LIPEC in obese animals when compared to their controls. In female rats (Fig. 4b), we observed that LIPEC induced similar accumulation of fat in WATs of both control and MSG groups, except for parametrial WAT, which had significantly increased fat levels in obese animals in comparison to non-obese ones.

## Discussion

The present study revealed that MSG-treatment induces obesity in male and female rats in different ways. It was shown that LIPEC in obese animals produced fat accumulation in visceral fat sites in a more accentuated manner in female than in male rats. In addition, we observed that among the visceral depots collected, the parametrial-WAT was the preferential one for lipid accumulation, indicating that fat mobility among WATs in lipectomized-obese rats can occur more expressively in specific depots of remaining WATs in a gender-dependent manner. However, the manner by which reminiscent territories of white adipose tissue behaves after LIPEC of subcutaneous WAT, as well as gender-related issues, is not yet completely understood. Therefore, in order to evaluate the effects of experimental LIPEC (parietal WAT removal) on different territories of adipose tissue in males and females we employed the well-known MSG-induced obesity rat model. MSG model has been extensively used in obesity studies and has contributed effectively to the understanding of different aspects of this disease. [18–24]

Initially, we observed that animals submitted to MSG treatment actually weighed less than control animals. These results are corroborated by previous studies performed by Kizer et al. (1977) [25], in which they show that MSG administered in the neonatal phase does not produce significant increase in body weight, when compared with non-treated animals. In this obesity model, the animals MSG-treated presented an imbalance in the hypothalamic-pituitary-adrenocortical (HPA) axis and among several endocrine changes, Ranke et al. (1988) [26] reported a decreased production of growth hormone (hyposomatotropism). Decreasing of naso-anal length was also previously described in this obesity model by Pizzi and Branhart (1976) [27] and Nemeroff et al. (1977) [23]. However, this lower weight gain cannot be attributed as secondary to a lower food intake, since these same authors previously reported that the animals MSG-treated presented normophagia.

The development of obesity exhibiting normophagia is rather intriguing, because MSG provokes damage in the arcuate nucleus of hypothalamus (ANH), an important area in the control of orexigenic and anorectic mechanisms [18, 21, 28–34]. Studies of Shi et al. (2007, 35] observed different approaches to reduce fat mass by caloric restriction or surgical fat removal (LIPEC) male and female rats affected food intake behavior, which can implicate directly in the capability to restore visceral fat. In the present study, we did not quantify food intake, as normophagia is a consolidated eating behavior in this model. On the other hand, the installation of obesity was observed on other parameters, such as increased total fat and adipose index.

In relation to the adiposity index, we observed that this parameter was significantly increased in both MSG-treated male and female rats. Surprisingly, the adiposity index was much higher (approximately 2-fold) in females than in males. These data clearly show marked gender-dependent differences in the accumulation of fats in MSG-treated animals. In addition, we observed that LIPEC in non-obese animals does not significantly alter the adiposity index, i.e., it does not induce obesity.

The total fats in the present study represent the sum of all WAT territories evaluated by us. In our study, this parameter permitted the evaluation of fat mobility in different WAT territories after LIPEC in obese animals. One of our main findings was the significantly higher visceral WAT accumulation in obese females than in obese males, which were submitted to LIPEC.

There are yet great controversy about the distribution and/or accumulation of lipids in the reminiscent WAT depots after LIPEC, as well as in relation the mechanisms involved, mainly when analyzed the influence of gender. Different from our findings, previous studies performed by Shi et al. (2007) [35] observed that female mice had a reduced capability to restore visceral fat (retroperitoneal depots) after fat loss by LIPEC. A possible reason for this discrepant result could be explained by the fact that we evaluate several visceral WAT depots and tried to evaluate the association of obesity and parietal LIPEC in the rat model. However, it is important to emphasize that we choose the withdrawal of parietal subcutaneous WAT depots because this has also been a routine in humans.

**Fig. 5:**
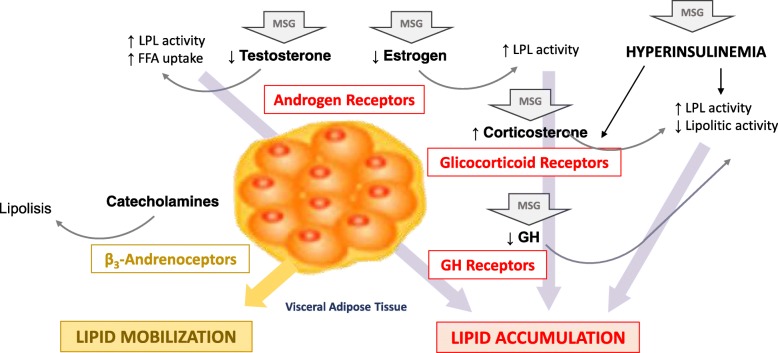
Mechanism of endocrine regulation of visceral adipose tissue in MSG-treated animals. Lipoprotein lipase, LPL; Free fat acid, FFA, monosodium glutamate, MSG and growth hormone, GH. MSG in arrows indicate hormonal changes observed in animals MSG-treated

When focusing the metabolic role of different regions of the body adipose tissue, cumulative data from the last decades of research consolidated the notion presented in 1947 by Vague [36] that obesity is not a homogeneous condition. Others have suggested that the data regional distribution of adipose tissue is important for the understanding of obesity and its correlation with metabolic disorders of glucose and lipids [37]. Several studies have shown that the harmful influence of obesity on metabolic processes is mediated by intra-abdominal fat deposition. In this sense, visceral fat has been correlated with glucose intolerance and associated with hyperinsulinemia during the oral glucose tolerance test, suggesting a clinical picture of insulin resistance [38–41] as has been evidenced by studies about the effects of neonatal MSG administration on insulin resistance. The increase in the adiposity of these animals was believed to be secondary to the increase in sympathetic hypothalamic activity, which decreases the transport of GLUT-4 in brown adipose tissue (BAT), consequently decreasing thermogenesis [42]. These effects could even be justified by the decrease of NPY levels in areas such as ANH and paraventricular nucleus (PVN) in the hypothalamus [43]. However, a more plausible hypothesis is related to glucose metabolism, since the increased sympathetic activity in visceral adipose depots seems too produce lipolysis and lipid mobilization by activating of β_3_-adrenoceptors (see Fig. 5). Previous studies in humans, showed that the lipolytic β_3_-adrenoreceptor sensitivity was 12 times higher in men, and the antilipolytic α_2_-adrenoreceptor sensitivity was 17 times lower in men, which lead us to conclude that in obesity, the catecholamine-induced rate of lipid mobilization from visceral fat is higher in men than women [44].

Nevertheless, the role of brown adipose tissue and its effects to thermogenesis cannot be ruled out. Adipocytes from obese patients are characterized by altered endocrine function, which leads to increased secretion of proinflammatory adipokines [45–47]. The activation of inflammatory pathways leads to insulin resistance in peripheral tissues, such as skeletal muscle and adipose tissue itself [48]. Only in the last decade, that skeletal muscle has been shown to behave an active endocrine organ that releases myokine, which is described as being able to communicate with cells in a local autocrine/paracrine manner within the muscles, or endocrine to distant tissues [49–51]. Myokines, peptides secreted by the muscles, are also produced by adipose tissue, activating thermogenesis in the brown adipose tissue in vitro and in vivo and participating in some metabolic functions and energy dissipation in these cells. Obese animals show higher expression of this peptide, suggesting some level of resistance to its effects [52, 53]. Perhaps, this could explain why, in the present study, LIPEC did not alter weight, total fat or adiposity index until the 16th week (when the animals were sacrificed) in non-obese animals, contrasting with what was observed in MSG-treated obese animals.

The novelty of the present study is the reporting of a significant increase in deposition the visceral WATs of lipectomized obese females compared non-lipectomized ones. To permit the discussion of our findings, we portrayed in Fig. 5 some possible mechanisms involved in the “Endocrine Regulation of Visceral Adipose Tissue” in MSG-treated animals.

Studies performed by Macho et al. (2000) [54] showed that early postnatal administration of MSG had an important effect on glucose metabolism and insulin action in adipocytes when the animals reached adulthood, where increases in insulin, glucose and leptin levels have been observed. Then, it would be plausible to assume that a possible hyperinsulinemia in MSG-treated animals could accentuate insulin lipogenic effects, inducing higher lipids synthesis and deposition into the adipocytes, contributing to the increased total fat and adiposity index observed in our study.

However, we believe that fat deposition in MSG-treated animals is more complex, and possibly due to multifactorial effects. In this regard, the effects of female hormones on WAT, particularly estradiol, should also be considered. Previous studies have shown that the weekly assessment of body weight revealed a significant increase in weight gain of ovariectomized females compared to non-ovariectomized females [55, 56]. It has been proposed by Naaz et al. (2002) [57] that adipose tissue is highly responsive to estrogen via receptors (ER) alpha and beta (ER-α and ER-β), with a more important role for ER-α in this tissue. Heine et al. (2000) [58] have previously suggested that estrogen regulates the amount of white adipose tissue through ER-α in an inhibitory way, and the absence of this receptor would cause adipocyte hypertrophy and hyperplasia, insulin resistance and glucose intolerance in both sexes. Birth studies conducted in 1991 reported that there was a sex-dependent response in the development of obesity through glutamate [59]. One of the possibilities would be due to obese females presenting a significant decrease in ovarian and uterine weight with consequent reduction in the number of primordial and Graafian follicles, with decrease of estradiol (E_2_) levels culminating in obesity and decreased body growth [60]. In fact, decreased levels of E_2_ have been found in MSG-treated obese rats [61]. In addition, E_2_ has shown a protective effect on insulin resistance [62, 63], as well as antidiabetic and auxiliary effects on body weight reduction even under a high-fat diet in rats [64]. Studies performed by Curi et al. (1991 [65] about neuroendocrine functions in neonatal MSG-treated rats evaluate possible alterations in lipogenesis rate and lipoprotein lipase (LPL) activity. These authors observed in female rats, increased lipogenesis in gonadal and retroperitoneal adipose tissues, while in male rats it was observed only in retroperitoneal adipose tissue. In addition, in female rats the administration of LPL inhibitor did not change retroperitoneal lipogenesis, suggesting that there is a specific sex-dependent response in the development of MSG-induced obesity. Although multifactorial, the extreme adipocyte mobility after LIPEC observed in our studies could be related to hypoestrogenism, as has been previously demonstrated in this model by Shen et al. (2014) [66].

On the other hand, the visceral adipose tissue has high density of androgen receptors and testosterone, which one can amplify its effect through upregulation of androgen receptors, inhibiting the expression of LPL and uptake of free fat acid (FFA). In men, visceral adipose tissue is negatively related to total and free plasma level of testosterone and by concentration of sex-hormone binding globuline (SHBG) [67]. Recent studies, have reported an interaction between glucocorticoid receptors and androgen receptors. The idea is that there is a “crosstalk” between these receptors in target tissues, like WAT and BAT. It was related that the androgen signal modulates transcriptional production of glucocorticoid receptors in a specific manner, i.e., the androgen receptor stimulation potentiated the transcriptional response to glucocorticoid receptor in vivo and in vitro in WAT and BAT, while the antagonism attenuated the signalization in both tissue [68]. Taking altogether, these studies appear to support the hypothesis that the deficiency of testosterone in male rats MSG-treated could contribute to accumulation of visceral adipose tissue. This low level of testosterone was demonstrated in studies performed by Ochiogu et al. (2015) [69].

Glucocorticoid receptors are considered those of the most important for human adipose tissue, and present an elevated density in visceral adipose tissue [70]. Previous studies reported by Bjorntorp, (1991) [71] showed that cortisol increases LPL activity and decreases lipolytic activity. In the Cushing’s disease, the important pathogenetic factor for visceral fat accumulation is observed the enhanced cortisol secretion resulting from a hyperactivity of HPA axis [72]. However, this increased lipid accumulation also depends, in addition to the excess of glucocorticoids, of the state of insulin resistance and decreased growth hormone (GH) levels [73]. In our study, all these changed levels hormones are present in MSG-model [26, 74–76], which could effectively induce to visceral lipid tissue accumulation. In addition, due to a major density of the glucocorticoid receptors are found in visceral than in other adipose tissues, is expected that the lipid-accumulating effect of cortisol would be more pronounced in visceral than in other fat areas [71].

The effects of GH on the regulation of visceral fat have to be considered separately. A decrease in circulating GH was previously demonstrated in human obesity and it was associated to an increase in visceral fat [71, 77]. Otherwise, a reduction in visceral adipose tissue was observed in humans with acromegaly, a state of GH excess [78]. Thus, most action of GH on adipose tissue are to prevent lipid accumulation and to stimulate lipid mobilization. However, the regulation of adipose tissue metabolism requires synergism with steroid hormones, and in relation to lipid accumulation, LPL is markedly inhibited by GH in the presence of either testosterone or cortisol [71]. Considering that their receptors are particularly dense in visceral adipose tissue, a more pronounced effect of GH would be expected in this tissue [71]. In this way, an important hallmark of MSG model is the GH deficiency, which, in turn, would be considered one of major factor to induce visceral fat accumulation, obviously in synergism to other changed hormonal factors above described.

Summarizing, low levels of estrogen/testosterone, deficiency of GH and increased secretion of corticosterone, which has been observed in MSG-treated rats, could induce higher visceral fat accumulation. In female rats MSG subjected to back of estrogen could justify why paramentrial-WAT is more responsive to lipid accumulation, as has been observed in women in postmenopause, when abdominal fat cell present high LPL activity.

The need to feel good about how we look is part of the human condition. Overweight and obesity usually lead the population to adopt miracle diets to lose weight or treat associated illnesses such as dyslipidemias and diabetes. On the other hand, “nutraceuticals” and “functional foods” has been frequently used as an alternative biological intervention to pharmacological methods [79–82]. Chen et al. (2008) [83] that reported the beneficial Influences of red mold rice (RMR) on obesity and dyslipidemia. Some evidences have been showed that Dietary polyphenols (DPs) are effective and promote health via multiple signalling pathways (such as lipid anabolism/catabolism pathways, apoptotic pathways) [84]. A combined treatment with genestein, quercetin, and resveratrol was shown higher inhibition of adipogenesis in both primary human adipocytes and 3 T3-L1 murine adipocyte than individual molecules [85]. In the near future, nutraceutical or functional foods, might be associated to pos-surgery procedures, as LIPEC, to prevent obesity, by the risk factor of health that could be entailed.

## Conclusions

The results of the present study led us to conclude that obesity induced by MSG treatment occurs differently in male and female rats. When associated with parietal LIPEC, there is a significant increase in the deposition of visceral fat, which is significantly higher in obese female than in male rats. Finally, these results allow us to infer that visceral WAT does not behave as a single tissue.

## Abbreviations

ANOVA: Analysis of variance
BAT: Brown adipose tissue
CNS: Central nervous system
CON: Control
EP: Epididymal
HAN: Hypothalamus arcuate nucleus
LIPEC: Lipectomy
MSG: Monosodium glutamate
OM: Omental
PM: Parametrial
PR: Perirenal
PVN: Paraventricular nucleus
RP: Retroperitoneal
SEM: Standart error mean
SHAM: Sham-operated surgery
WAT: White adipose tissue
